# The Role of Job Demands–Resources (JDR) between Service Workers’ Emotional Labor and Burnout: New Directions for Labor Policy at Local Government

**DOI:** 10.3390/ijerph15122894

**Published:** 2018-12-17

**Authors:** Sunhee Kim, Jaesun Wang

**Affiliations:** 1 Department of Public Administration, Seowon University, Musimseoro 377-3, Cheongju 28674, Korea; shkim7675@hanmail.net; 2 Department of Public Administration, Honam University, 417, Eodeung-daero, Gwangsan-gu, Gwangju 62399, Korea

**Keywords:** emotional labor, burnout, job demands–resources model

## Abstract

Recently, research on service workers’ emotional labor has received considerable attention, both in theory and practice. Emotional labor has been reported to cause both stress and burnout in service workers, eventually leading to a decrease in organizational productivity. In this context, there is also a growing interest in identifying ways to reduce such burnout. This study aimed to examine the influence of emotional labor and job demands–resources (JD–R) on service workers’ burnout. Specifically, we analyzed the direct, indirect, and moderating effects of JD–R on burnout. Data were collected from service workers (*N* = 1517) in public sectors. Results revealed that three dimensions—emotional labor, intensity/variety, and surface acting—increase burnout, whereas deep acting decreases it. Additionally, job demands were found to increase burnout, while job resources decreased it. Among the job demands, customer contact had the greatest positive impact on burnout, followed by role ambiguity and workload, respectively. Among the job resources, self-efficacy and social support had the greatest negative impact on burnout. Finally, customer contact, role ambiguity, job autonomy, and social support were identified as moderators that worsened or buffered the impact of emotional labor on burnout.

## 1. Introduction

As the service industry grows, the number of service workers and their importance have been increasing. Since the quality of the service provided is the key comparative competitiveness advantage of service organizations, service management prioritizes the enhancement of service quality, and organizations focus on management skills pertaining to the service. Accordingly, organizations attempt to control and regulate the emotions that workers exhibit to customers, in an attempt to enhance service quality. Service work is mainly processed by individuals. Service workers include flight attendants, office assistants, clerks, teachers, police officers, nurses, salespeople, call-center employees, etc. Those in the service field usually have intensive interactions with customers, process their requests, and produce outputs while mobilizing their emotional resources. Brotheridge and Grandey [1] explained that “people work” is one of the main characteristics of service labor. They found significant differences in the nature of prototypical emotional occupations and burnout jobs, as well as the existence of a hierarchy of emotional labor expectations and demands. 

The growth of the service sector has increased the academic interest in workers’ labor content. Several studies have attempted to determine factors affecting service workers’ behavior. Among these factors, researchers have paid attention to emotional labor not only as a managerial issue but also as a theoretical one. Hochschild [2] was the first sociologist to define the concept of emotional labor, describing it as a form of emotion regulation that creates a publicly visible facial and bodily display within the workplace [1].

Emotional labor is a social construct created through interactive relationships between workers and customers. This suggests that emotional labor can be formed through social exchange; workers can influence customers and be influenced by social relations with them. Based on the interaction model of emotional labor, emotional work is often subject to external forces [3,4]. In her seminal book published in 1983, Hochschild analyzed flight attendants and reported that their work could not be fully defined by the physical aspects and cognitive demands alone, but that we must consider the emotions they were required to express while dealing with passengers. 

Emotion work can create positive outputs for employers. It helps render social interactions more predictable, avoid embarrassing situations, and develop trust in the organization, thus developing or stabilizing the relationship between the organization and customer [5]. Emotion work is characterized by the following features: (1) it occurs in face-to-face or voice-to-voice interactions with clients; (2) the emotions displayed influence other individuals’ emotions, attitudes, and behaviors; and (3) the display of emotions has to follow certain rules [2,5,6,7]. 

However, emotional labor creates negative byproducts such as personal-role conflict, ego depletion, felt inauthenticity, inauthentic expression of emotion, negative emotion, impairment of social interaction in intermediate process and emotional exhaustion, depersonalization, lack of personal accomplishment, psychological strain, and psychosomatic complaints in consequence [8]. Many studies have focused on various negative consequences of emotional labor [1,4]. Accordingly, it is important to identify ways to reduce such negative outcomes; however, few studies have done so. Recently, Maslach and Goldberg [8] focused on ways to reduce burnout through increasing engagement and reframing the concept of burnout itself. To understand the healing potential of emotional labor, one must examine the moderating factors that intervene in the relationship between emotional labor and its negative outcomes, e.g., burnout. Moreover, there is an inconsistent relationship between emotional labor and burnout. Although Brotheridge and Grandey [1] reported a positive association between surface acting and burnout, there was no association between the intensity/variety of emotional labor, deep acting, and emotional exhaustion. Such inconsistencies can be explained by other intervening variables. Moreover, after Morris and Feldman [4] conceptualized emotional labor as a multidimensional construct, they demanded that the related research should examine potential moderators and/or mediators in the relationship between emotional labor and psychological wellbeing. After that, there have been lots of studies examining such moderating and/or mediating effects by adopting other variables.

Accordingly, the present study aimed to identify ways to reduce the burnout caused by emotional labor. First, we examined how emotional labor influences service workers’ burnout. Second, we analyzed whether job demands and resources (JD–Rs) relieve or worsen burnout. Finally, we analyzed the direct and indirect moderating effects of JD–R on burnout. We expect that this study will highlight ways to reduce the emotional exhaustion caused by emotional labor.

## 2. Theory and Hypotheses

### 2.1. Emotional Labor

According to Hochschild emotion work refers to “the act of trying to change the degree or quality of an emotion or feeling” ([3], p. 561). Emotion work differs from emotion “control” or “suppression.” The latter two terms suggest an effort to merely stifle or prevent an emotion. Emotion work, however, refers more broadly to the act of evoking or shaping, as well as suppressing, one’s emotions ([3], p. 561). Moreover, Morris and Feldman [4] defined emotional labor as the effort, planning, and control needed to express organizationally desired emotions during interpersonal transactions. According to Grandey [9], emotional labor is the process of managing feelings and expressions to fulfill the emotional requirements of a job. 

Hochschild identified the following types of emotional work: cognitive, bodily, and expressive ([3], p. 562). In cognitive emotion work, one attempts to change images, ideas, or thoughts to change the associated feelings. It thus involves recodification, in which a situation is reclassified into the previously established mental categories of a situation. In bodily emotion work, one attempts to change somatic or other physical symptoms, e.g., trying to breathe slower, trying not to shake. In expressive emotion work, one tries to change expressive gestures in an attempt to change inner feelings, e.g., trying to smile or cry. These three types of emotion work often occur simultaneously. 

Studies on emotional labor have employed the following three perspectives: dimensions, antecedents, and consequences of emotional labor. Earlier, Morris and Feldman [4] suggested the following four dimensions of emotional labor: frequency of appropriate emotional display, attentiveness to required display rules, variety of emotions to be displayed, and emotional dissonance generated by having to express organizationally desired emotions that are not genuinely felt. Zapf [5] also focused on four dimensions; automatic emotion regulation, where required emotions are spontaneously experienced by the employees; surface acting, where employees make efforts to manage the visible aspects of emotions that appear on the surface, which generates a conflict between their inner feelings and their outer expressed behaviors because the inner feeling remains unchanged; deep acting, where employees try to influence what they feel in order to take the role they are required to display; and emotional dissonance, which appears when an employee is required to express emotions that are not genuinely felt in a given situation. Moreover, Grandey [10] suggested two dimensions of emotion work, emotional dissonance and emotional effort.

Studies on the antecedents of emotional labor have focused on the influence of organizational, job-related, and individual factors that affect emotional labor. Morris and Feldman [4] included explicit display norms and close employee monitoring at the organizational level; job autonomy, forms of interaction, and task variety/routineness at the job level; and affectivity and gender at the individual level. 

Studies on the consequences of emotional labor focused on the negative byproducts of emotional labor. Morris and Feldman [4] hypothesized that, among the four dimensions of emotional labor, only emotional dissonance leads to higher emotional exhaustion and lower job satisfaction. Brotheridge and Grandey [1] reported that surface acting diminished workers’ sense of personal accomplishment, whereas deep acting contributed to a greater sense of efficacy. Our study focused on burnout as a consequence of emotional labor. 

### 2.2. Burnout

After observing volunteer workers in New York, Freudenberger used the term “burnout” to describe their emotional depletion and loss of motivation. Freudenberger defined burnout as “a state of mental and physical exhaustion caused by one’s professional life,” and as “the extinction of motivation or incentive, especially where one’s devotion to a cause or relationship fails to produce the desired results” ([11], p. 159). Generally, individuals who experience burnout in their work drain out their energy resources and lose their work engagement. According to Maslach and Jackson [12], after human services professionals in California spent considerable time in intense service work involving interacting with clients, they experienced negative feelings such as anger, embarrassment, fear, or despair. Further, continuously working under such circumstances increased their risk of burnout—emotionally drained chronic stress. Maslach and Jackson [11] proposed that burnout is a syndrome comprising the following three subcomponents: emotional exhaustion, depersonalization, and the feeling of a lack of personal accomplishment. Emotional exhaustion refers to feelings of being emotionally drained by one’s contact with other individuals ([13], p. 390). When service workers feel that their emotional resources have depleted, they are no longer able to engage in the work. Depersonalization refers to a negative or excessively detached response toward the service or service recipients ([13], p. 390). It includes the negative and cynical attitudes or feelings toward customers. Schaufeli et al. [14] substituted the term “depersonalization” for cynicism, which refers to a distant attitude toward work in general, not necessarily directed toward other individuals. Finally, reduced personal accomplishment refers to a decline in one’s feelings of competence and successful achievement at work ([13], p. 390). It is the negative evaluation of oneself as a service worker, and in particular, involves negative feelings over one’s interactive work with service customers. 

Burnout is an output of emotional labor. Burnout researchers have only recently started integrating emotion work into the concept of burnout ([5], p. 256). Maslach [15] argued that interactions with clients are often difficult and upsetting because health professionals face troubled individuals. Frequent face-to-face interactions with those clients required longer periods of intensive emotional effort, which were related to higher levels of emotional exhaustion. Morris and Feldman [6] found a significant negative correlation between the frequency of emotion work and exhaustion. Additionally, Zapf et al. [16] found that the requirement to display particular emotions was positively correlated with emotional exhaustion. Moreover, Brotheridge and Grandey [1] reported a positive association between intensity/variety, surface acting, and burnout. According to Grandey [17], surface acting (modifying facial expressions) and deep acting (modifying inner feelings) performed contrasting roles. Specifically, while the former was negatively related to affective delivery, the latter was positively related to it. Moreover, the former, not the latter, is related to stress. Similarly, surface actors influence exhausted and cynical burnout more positively than deep actors do [18]. Hochschild [2] proposed that surface acting may create dissatisfaction, whereas deep acting may enhance the sense of satisfaction. According to Scott and Barnes [19], affective states worsened when employees engaged in surface acting, while they improved when they engaged in deep acting. Moreover, surface acting significantly increased work withdrawal. Moreover, Diefendorff et al. [20] demonstrated that unit-level display rules have associated with burnout indirectly by ways of display rule perceptions at the individual-level and emotion regulation strategies. Based on these prior findings, we proposed the following research hypotheses:

H1: The intensity/variety of emotional labor is positively related to burnout.

H2: Surface acting is positively related to burnout, whereas deep acting is negatively related to it.

### 2.3. The Job Demands–Resources Model

The JD–R model, developed by Bakker et al. [21] and Demerouti et al. [22], proposes that working conditions can be divided into two broad categories, job demands and job resources, which are differently linked to specific performances in the workplace. Demerouti et al. defined job demands as those physical, social, or organizational aspects of the job that require sustained physiological and psychological costs (e.g., exhaustion) ([22], p. 501). It is a kind of stressor that, as an external factor, has a negative impact on workers. Job resources refer to those physical, psychological, social, or organizational aspects of the job that may fulfill any of the following roles: (a) be functional in achieving work goals; (b) reduce job demands together with their associated physiological and psychological costs; or (c) stimulate personal growth and development (p. 501). Demerouti et al. [22] focused more on external resources (organizational, social) than internal ones (cognitive features and action patterns) because the former can be regarded as stable or situationally independent and manageable by job design. If workers lack resources, they cannot cope with the negative impact of demands, and they would finally fail to achieve the goal. 

Based on the JD–R model, Demerouti et al. [22] proposed that burnout follows two processes. First, the demands of work, e.g., physical workload, time pressures, recipient contact, physical environment, and shift work, lead to exhaustion. Second, a lack of job resources, e.g., feedback, rewards, job control, participation, job security, and supervisor support, lead to disengagement. Similarly, Bakker et al. [13] found that, in the JD–R model, the two processes operate independently. The energy-driven process is “job demands → burnout → negative performance,” whereas the motivation-driven process is “job resources → engagement → positive performance”.

Job demands and job resources are not measured by themselves but by other sub-variables that represent them. As dimensions of job demands, Lee and Ashforth [23] suggested that ambiguity/clarity, task overload, role conflict, role stress, stress incidents, work pressure, and so on, play major roles. Additionally, Demerouti et al. [22] and Alarcon [24] suggested role ambiguity, conflict, and task overload as variables to represent physical workload, time pressures, customer contact, the physical environment, and shift work. 

In contrast, Christian et al. [25] found that job variety, meaningfulness, autonomy, feedback, social support from peers, close relationship with one’s supervisor, and transformational leadership were components of job resources. Similarly, Demerouti et al. [22] focused on feedback, compensation, job control, participation, job security, and supervisor support as aspects of job resources. Moreover, Lee and Ashforth [23] found that social supports (job support, peer support, work friends, community groups, family resources, peer cohesion, and team cohesion), change in job enhancements (autonomy, innovation, participation, use of technology, and task orientation), and reinforced situations (unmet expectations, situation-contingent compensation, and punishment) were significant subcomponents.

The critical systematic relationship between job demands/resources and outcomes at the individual and organizational level has been observed in previous studies. In the meta-analysis conducted by Lee and Ashforth [23], job demand variables, as compared to job resources, were found to be important predictors of exhaustion. The meta-research conducted by Christian et al. [25] reported that job resources are the most important predictor of employees’ emotion. 

The key theoretical proposition in the JD–R model is that job demands are primarily responsible for burnout, and that job resources primarily influence enthusiasm. Demerouti et al. [22] verified this model through empirical studies. Schaufeli and Bakker [26] confirmed that job demands and resources are negatively related to each other, while job demands are related to exhaustion and job support affects enthusiasm. Additionally, the influence of job resources on burnout is smaller than that of job demands is. Recently, based on a meta-analysis, Alarcon [24] reported that higher demands and lower resources are associated with burnout. Moreover, there are intersections between job demands and resources. Bakker et al. [27] explained that burnout comes from an imbalance between job demands and resources. They reported that resources play a role in buffering the impact of several job demands on burnout. Therefore, we expect that job demands increase burnout, whereas job resources decrease it. 

### 2.4. Job Demands and Burnout

#### 2.4.1. Role Ambiguity

*Direct Effect:* Role ambiguity is contrasted with task routineness. Higher task routineness is negatively associated with emotional labor, particularly with attentiveness to required emotion display rules [4]. Based on their study on 469 teachers, Schwab and Iwanicki [28] reported that role ambiguity explains much of the variance in burnout, specifically in the form of depersonalization and exhaustion. Similarly, based on a study on 562 teachers, Papastylianou et al. [29] demonstrated that role ambiguity shows a significant negative correlation with emotional exhaustion and depersonalization. Tunc and Kutanis’ [30] examination of 251 healthcare professionals (170 physicians and 81 nurses) at a university hospital in Turkey revealed that role ambiguity explains the higher level of burnout experienced by the nurses as compared to the physicians.

A meta-research by Lee and Ashforth [23] and Alarcon [24] reported that role ambiguity is positively associated with burnout and its subcomponents. Brooking et al. [31] demonstrated statistically significant relationships between role ambiguity and all three burnout components in 135 female human service professionals.

H3-1: Role ambiguity is positively related to burnout.

*Moderating Effect:* Based on a literature review on emotional labor, Hülsheger and Schewe [7] suggested that the significant effect of emotional labor (rule dissonance) on emotional exhaustion and depersonalization is moderated by person–role conflicts. Moreover, on examining burnout in police officers, Kwak et al. [32] reported that emotional labor and role stressors (role ambiguity and conflict) are related to police officers’ greater burnout. According to Tetrick et al. [33], based on survey data from one hundred sixty licensed morticians, perceived lower levels of role ambiguity and role conflict decreased the level of emotional exhaustion, whereas they increased the levels of job satisfaction and professional satisfaction. Based on these findings, we proposed the following hypothesis:

H3-2: Role ambiguity will moderate the relationship between emotional labor and burnout.

#### 2.4.2. Workload

*Direct Effect:* Work overload is a dimension of job demands. It is positively associated with burnout, particularly with exhaustion [34]. Lee and Ashforth [23] reported that task overload is positively correlated with emotional exhaustion. Based on their study on 1363 nurses employed in hospitals, Greenglass et al. [35] found that emotional exhaustion increases cynicism and somatization, and that cynicism had a negative relationship with nurses’ professional efficacy. Further, by analyzing survey data from a sample of 357 registered nurses, licensed practical nurses, and non-registered caregivers from two psychiatric hospitals in Belgium, Van Bogaert et al. [36] found that workload plays a major role in accounting for job outcomes, ultimately explaining 60% of the variance in these variables.

H4-1: Workload has a positive relationship with burnout.

*Moderating Effect:* Moreover, work overload can aggravate the negative effects of emotional labor because it has a positive relationship with emotional labor and burnout. Morris and Feldman [4] assumed that task variety and routineness are associated with emotional labor, and ultimately, with burnout. In a meta-analysis, Demerouti et al. [22] found positive relationships between physical workload and exhaustion. Likewise, Alarcon’s meta-analysis showed that task overload is positively associated with burnout [24]. Higher work demands, which require the management of diverse individuals, functions, and lines of business, intervened these relationships between emotional intelligence and team effectiveness [37]. Moreover, the quality or kind of work affects burnout. When service providers viewed their tasks as more challenging, the requirement to engage in deep acting was less emotionally exhausting [38].

H4-2: Workload will moderate the relationship between emotional labor and burnout.

#### 2.4.3. Customer Contact

***Direct Effect:*** Service workers are usually responsible for making contact with customers. In Demerouti et al.’s JD–R model, customer contact is one of significant variables that influence burnout—particularly, exhaustion and disengagement [22]. Based on a survey on 34 occupational therapists (response rate approximately 60%) in a metropolitan public mental health service, Scanlan and Still [39] reported that the respondents’ contact demand (the perception that contact with service users or families was demanding) was the most significant variable associated with poorer wellbeing (higher turnover intention and burnout). Cordes and Dougherty [40] suggested that interaction between customers’ roles and suppliers’ expectations in the service process could cause burnout.

Customer contact itself did not lead to an increase in burnout. When customer revealed more negative and aggressive attitudes and actions toward service workers, the contact worsened their wellbeing. Kim and Park [18] reported that customer-related social stressors (CSS) are the most significant variables that influence burnout. They reported that CSS was associated more strongly with burnout than with engagement. In a study on 198 call center employees, Grandey et al. [41] found that the frequency and stress appraisal of customer aggression increases burnout. Moreover, employees who experienced more threats through customer aggression tended to mobilize emotional labor as a defense mechanism. Among the different types of customer contact, in the present study, we focused on aggressive customers.

H5-1: Negative customer contact is positively related to burnout.

*Moderating Effect:* Côte’ [42] examined interpersonal processes that may explain how the emotional labor of service workers is evaluated by customers, who in turn respond accordingly and thereby influence the employees’ emotional and psychological health. Moreover, based on data from 120 bank tellers, Sliter et al. [43] reported that customer incivility was positively related to emotional exhaustion.

H5-2: Negative customer contact will moderate the relationship between emotional labor and burnout.

### 2.5. Job Resource and Burnout

#### 2.5.1. Self-Efficacy

*Direct Effect:* Resources could influence the relationship between job demands and burnout. Bakker et al. [27] explained that several job resources may compensate for the impact of several job demands on burnout. Self-efficacy, which is one of these resources, has the most significant influence on burnout. Self-efficacy generally reduces burnout. 

In a review of 57 studies on the relationship between burnout and self-efficacy, Shoji et al. [44] found that the size of the estimated effect of the relationship between self-efficacy and burnout is moderate (−0.33). However, it depends on the subject of study; it is higher in teachers, older workers, and workers with long-term experience than in medical care workers. Schwarzer and Hallum [45] reported that self-efficacy has a negative effect not only on current burnout but also on future burnout. On investigating the association between emotional labor and burnout, Jeung et al. [46] found that emotional labor is a job stressor that leads to burnout. Among personality traits, self-efficacy and type A behavior patterns influence burnout. In a study on 244 Norwegian teachers in elementary and middle school, Skaalvik and Skaalvik [47] found that self-efficacy mediates their perceived collective efficacy, external control, and burnout.

H6-1: Self-efficacy is negatively related to burnout.

*Moderating Effect:* Van Yperen [48] demonstrated the moderating effect of self-efficacy in the relationship between informational support, equity, and burnout. Moreover, Jimmieson [49] reported that self-efficacy moderated the main effects of work control on job satisfaction and somatic health. Based on survey data from public service employees in Taiwan, Hsieh et al. [50] reported that self-efficacy mediates the relationship between emotional labor and job satisfaction. Job satisfaction is other side of burnout. Accordingly, we hypothesized that self-efficacy may reduce the negative effect of emotional labor on burnout.

H6-2: Self-efficacy will moderate the relationship between emotional labor and burnout.

#### 2.5.2. Job Autonomy

*Direct Effect:* Hackman and Oldham [51] defined job autonomy as the degree of freedom, independence, and discretion which an employee has in accomplishing job tasks. Job autonomy tends to influence organizational effectiveness and job satisfaction positively. Morris and Feldman [4] regarded job autonomy—in particular, emotional dissonance—as an important antecedent of emotional labor since employees with more autonomy are able to violate organizational rules when they conflict with their own emotions. Consequently, they experience less emotional labor. 

A study by Jackson et al. [52] reported that autonomy has a negative relationship with emotional exhaustion and depersonalization. Moreover, Lee and Ashforth [23] reported that work autonomy has a negative relationship with the sub-dimension of exhaustion, which, in turn, is strongly related to emotional exhaustion, followed by depersonalization and personal achievement, respectively. According to the meta-study conducted by Alarcon [24], autonomy is negatively associated with emotional exhaustion and cynicism.

H7-1: Job autonomy is negatively related to burnout.

*Moderating Effect:* Bakker et al. [27] reported that job autonomy was the resource that acted most often as a buffer for emotional demand. On the other hand, Han et al. [53] reported that emotional labor has a positive impact on depressive mood in service and sales workers. However, such effects are moderated by job autonomy; high emotional labor was associated with depressive mood only in the presence of low job autonomy in male workers. Based on a meta-analysis, Humphrey et al. [54] reported that emotional labor may have positive outcomes when organizations grant more autonomy and adopt positive display rules that call for the expression of positive emotions.

H7-2: Job autonomy will moderate the relationship between emotional labor and burnout.

#### 2.5.3. Social Support

*Direct Effect:* Lee and Ashforth [23] reported that social support is negatively associated with the sub-dimension of exhaustion, especially with emotional exhaustion. Moreover, they reported that the support of superiors has less power to reduce burnout than the support of peers does. Jackson et al. [52] confirmed this effect of superiors’ support. Demerouti et al. [22] reported a significant positive correlation between supervisor support and exhaustion. Zapf [5] reported that social support moderates the relationship of emotion work variables with burnout and job satisfaction

H8-1: Social support is negatively related to burnout.

*Moderating Effect:* Interpersonal relationships, including social support and relationships at the workplace, create an environment that reduces burnout. For example, Park et al. [37] demonstrated that interpersonal influence training might be beneficial to reduce the negative effect of surface acting among school employees. Morris and Feldman [4] suggested that social support is a moderator in the relationship between emotional labor and psychological wellbeing. Bakker et al. [27] also reported that social support from colleagues, and the quality of workers’ relationships with their supervisor, could buffer the effects of emotional demand. Day et al. [55] found that, when organizational change stressors were associated with higher burnout, social support buffered the relationship between change stressors and burnout, i.e., exhaustion and cynicism. According to Tetrick et al. [33], less social support from work-related sources induced less emotional exhaustion. Moreover, Jawahar et al. [56] demonstrated that perceived organizational support was associated with less emotional exhaustion and depersonalization, and it moderated the relationship between role conflict and emotional exhaustion. Hülsheger and Schewe [7] suggested that surface and deep acting will influence personal ill-being, job-related wellbeing, and performance, through the enhancement or impairment of social interaction.

H8-2: Social support moderates the relationship between emotional labor and burnout.

#### 2.5.4. Job Resource as Moderator for Job Demand

The JD–R model predicts that job resources decrease the negative effect of job demands on exhaustion [57]. Bakker et al. [58] reported that the impact of job demands on exhaustion was especially strong if employees had less job resources. Similarly, the effect of job resources on cynicism was particularly effective if employees faced many job demands. In a subsequent study, Bakker et al. [58] found that all possible interactions between individual job demands and job resources (autonomy, social support, and quality of relationship with supervisors) were significant, in the hypothesized direction of impact.

H9: Job resources moderate the relationship between job demand labor and burnout.

**Figure 1:**
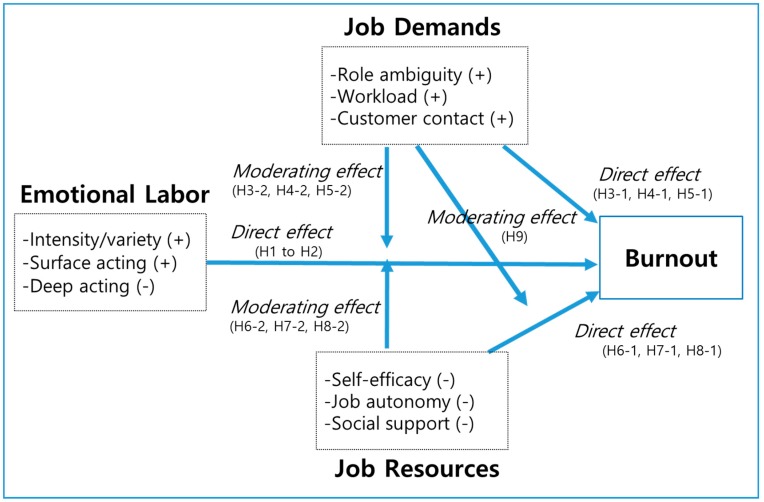
The research framework.

Figure 1 presents the research framework.

## 3. Sample and Measures

This study analyzed survey data collected from service workers in public sectors, i.e., firefighters, administrative officers, nurses, and police officers, between 1 April 2016 and 30 June 2016. All of them were employed under province of GyeongGi-do, a large regional government in Korea. Participants were selected by using random stratified sampling. In total, 1800 questionnaires were distributed and 1590 (88.3%) were collected. After excluding questionnaires with poor answers, 1517 cases were used for the analysis.

Detailed information on the sample and sampling method for the four types of organizations included in this study is presented in Table A1. Using quota and stratified random sampling, we selected targeted offices from each organization. We then recruited an internal collaborator in each of the four organizations to seek help and monitor the survey process. The questionnaire was distributed after the internal collaborator asked candidates for respondents whether they were willing to participate in the survey. If they were not, other potential respondents were asked. We obtained informed consent from all participants. The respondents completed the questionnaire on their own and submitted it to the survey distributor. 

According to the article of the Bioethics and Safety Act (enacted in 2005) in Korea, which defined the exemption approval of a review committee in case of surveys not involving face-to-face interviews. Since the present study did not involve face-to-face interview, the review was exempted. The questionnaire was distributed to the respondents, after which they completed it alone and returned it to the distributor. Moreover, the present study did not collect personal information, the requirement for review was waived.

This study adopted the burnout measure developed by Schaufeli et al. [14] based on the Maslach Burnout Inventory—General Survey (MBI–GS). This tool comprises items on emotional exhaustion, cynicism, and inefficacy. Since a factor analysis divided these three components into two factors (the first factor includes both emotional exhaustion and cynicism, whereas second one covers inefficacy), we used the mean score on the two variables included in the first factor. 

Additionally, this study utilized the emotional labor assessment tool developed by Brotheridge and Lee [59]. This tool assesses the following four dimensions of emotional labor: intensity, variety, surface acting, and deep acting. Emotional intensity refers to how strongly or with what magnitude an emotion is experienced or expressed ([4], p. 1990). Emotional variety refers to the number of different emotions required to be expressed. Emotional labor increases when employees are expected to express a variety of emotions. Surface acting refers to the efforts invested in managing the visible aspects of emotions that appear on the “surface,” whereas deep acting refers the efforts spent in regulating deeply felt emotions ([5], p. 244). The factor analysis conducted in the present study revealed that intensity and variety could be included in the same factor (see Table 1). Therefore, we used the mean score on all variables for these two factors. 

We did not translate the English measurement instruments on emotional labor and burnout because they had already been validated by Lee et al. [60] and Shin [61], respectively. Specifically, Lee et al. [60] revised the Emotional Labor Scale developed by Brotheridge and Lee [59], while Shin [61] revised the burnout scale developed by Schaufeli et al. [14].

**Figure 2:**
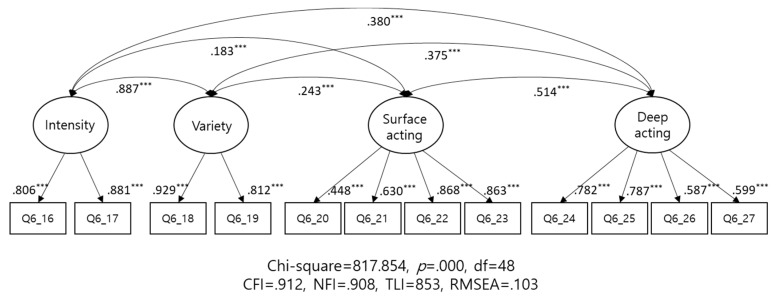
The four factor model. Note: * *p* < 0.05; ** *p* < 0.01; *** *p* < 0.001.

**Figure 3:**
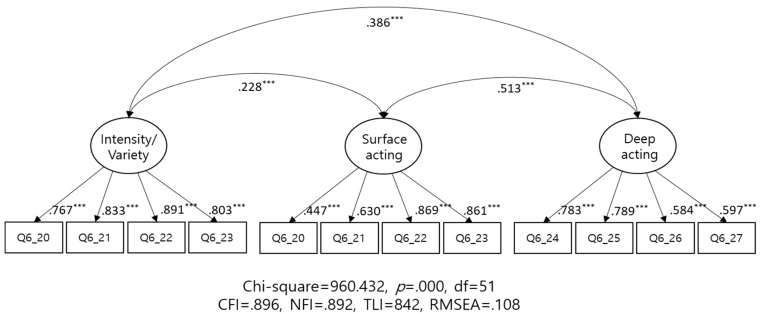
The three factor model. Note: * *p* < 0.05; ** *p* < 0.01; *** *p* < 0.001.

To confirm convergent and discriminant validity of emotional labor scale, we first conduct a factor analysis, as shown in Table 1. The principle component analysis conducted with varimax rotation yielded three factors. Subsequently, we checked the convergent and discriminant validity using structural equation modelling, and we compared the findings for the three- and four-factor structures of the scale. As evident from Figure 2 (for the three-factor model) and Figure 3 (for the four-factor model), both models were statistically significant. Convergent validity was determined by (1) a standardized factor loading of 0.5 between the latent and measurement variables, (2) a construct reliability (CR) of 0.7, and (3) an average variance extracted (AVE) of 0.5. The two models satisfied three criteria, i.e., factor loading, CR, and the AVE (excepting deep acting). Interestingly, the CR for intensity/variety increased from 0.832 to 0.864 in the four-factor model and to 0.895 in the three-factor model. This implies that the three-factor model could be a more reliable measure as compared to the four-factor model.

To confirm discriminant validity, the AVE value is required to be larger than that of the correlation squared. As evident from Table 2, the three-factor model satisfied this criterion, while the four-factor model did not. Regarding the correlation between latent variables, a higher correlation (0.887; Table 2) was observed between intensity and variety, indicating the low discriminant validity of the subscales measuring these two factors. Moreover, the low correlation coefficients between intensity and variety, and between surface and deep acting confirmed a significant degree of discriminant validity between the latent variables. However, the four-factor model exhibited better values on the goodness-of-fit indexes than the three-factor model did. 

Further, to assess components of JD–R, we used the role ambiguity measure developed by Rizzo et al. [62], the workload scale developed by Leiter and Maslach [63], the self-efficacy scale developed by Shim and Ha [64], the job autonomy scale developed by Breaugh [65], and the social support scale developed by Grandey [66]. Negative customer contact was assessed using the CSS scale developed by Dormann and Zapf [67]. The reliability statistics for all these scales are presented in Table 1. 

To check for the common method bias, we applied the Herman test proposed by Padsakoff and Organ [68]. A confirmatory factor analysis on the dependent and independent variables revealed that the variance explained by one factor was 17.47%. Thus, the presence of common method bias was not considered serious.

## 4. Analysis and Findings

### 4.1. Descriptive Analysis

To determine the associations between variables, we examined only simple but also partial correlations which controlled age, income, education, and social class. The results are presented in Table 3, in which values above the diagonal line represent the partial correlation coefficients, whereas those below the diagonal represent Pearson’s correlation coefficients. 

Values in the second to the fourth row and the third to fifth column represent the correlations and partial coefficients for the three dimensions of emotional labor. The moderate values, i.e., 0.247, 0.314, and 0.390, demonstrate that each dimension of emotional labor has a relationship with other variables, but they maintain an independent arena of variance. 

Emotional labor had positive relationships with all the variables of job demand. However, the association changed according to the subcomponents of job demand. Specifically, role ambiguity had the strongest correlation with surface acting, a moderate association with intensity/variety, and the weakest association with deep acting. Further, variety had the strongest correlation with workload, followed by surface and deep emotional labor, respectively. Finally, customer contact had the strongest correlation with surface emotional labor, followed by deep emotional labor and intensity/variety, respectively. 

Notably, each of three dimensions of emotional labor had a different relationship with the job demand variables. Specifically, deep acting had the strongest correlation with workload, while surface acting had the strongest correlation with customer contact. On the other hand, intensity/variety had the weakest association with customer contact, as did surface acting with workload, and deep acting with role ambiguity. These findings implied that it is important to consider various demand factors to help reduce emotional labor. For example, if managers want to reduce intensity/variety, they should decrease the employee’s workload, whereas if they wish to lower surface acting, they should consider reducing the amount of customer contact. 

The present analyses also revealed that emotional labor had a positive association with the dimensions of job resources. Specifically, intensity/variety and deep acting had a positive relationship with self-efficacy and job autonomy. Moreover, deep acting had a positive association with social support. This result is noteworthy because it is generally expected that positive resources can reduce emotional labor. However, surface acting had no significant relationships with job resources. 

Emotional labor was also positively related to burnout; however, the relationship varied according to the dimensions of emotional labor. Specifically, intensity/variety and surface acting had significant relationships with burnout, while deep acting did not exhibit such an association. Additionally, surface acting appeared to have a stronger correlation with burnout than intensity/variety did. These results confirm the findings of a previous study [1] that surface acting and burnout were positively correlated and that surface acting had the strongest correlation with emotional exhaustion. However, that study did not find a significant relationship between the intensity/variety of emotional labor, deep acting, and burnout. 

The present study also showed that burnout had a positive relationship with job demands, but a negative relationship with job resources. Further, among the dimensions of job demands, burnout had a relatively stronger correlation with job ambiguity and customer contact than with workload. Similarly, Demerouti et al. [22] reported positive relationships between physical workload/customer contact on one hand, and exhaustion and disengagement on the other. 

Burnout was also found to exhibit a relatively stronger association with self-efficacy and social support than with job autonomy. This result suggests that workers themselves are more important than the job design is, and that social relationships in the workplace play a significant role in reducing burnout. According to Demerouti et al. [22], supervisor’s support has a positive relationship with exhaustion. 

Moreover, job demand variables had stronger correlations with burnout than did job resource variables. This confirmed the findings of the research conducted over the past decade, which revealed that job resources are less strongly related to burnout than job demands are [13].

### 4.2. Regression Analysis

To identify the predictors of burnout, we conducted a regression analysis with emotional labor, job resources, and job demands as explanatory variables. Model 1 through 4 show the structure and explanatory power for each of the four factors in predictors, while Model 5 presents the full model. The results are presented in Table 4. 

Model 1 shows how the sociodemographic variables such gender, age, year of employment, position in the organization, household income, and education influenced burnout. Six demographic variables accounted for merely 5.2% of the variance in burnout. Among these six variables, three predictors—sex, position in the organization, and household income—showed a significant impact on burnout. 

Further, individuals at a higher position in the organization experienced more burnout. Such individuals often assume more responsibility for the organization and their work. Therefore, they usually experience a greater burden, which leads to burnout. However, Cordes and Dougherty [40] reported that those in a higher position in their career may experience less burnout because promotions tend to decrease client contact. 

Higher income significantly increased burnout. This may be because it is assumed that, obtaining a higher income requires more emotional effort, eventually leading to a higher level of burnout. However, age, year of employment, and education level did not have a significant impact on burnout in the present study, which is in contrast to previous findings reported by Ahola et al. [69] pertaining to age and work experience, and those of Cordes and Dougherty [40] regarding educational level.

In Model 2, the three dimensions of emotional labor were examined as predictors of burnout. Those who experienced higher emotional labor tended to experience a higher level of burnout. All three dimensions had a significant impact on burnout, in contrast to the findings reported by Brotheridge and Grandey [1], that intensity and variety of emotional labor did not influence any of the three dimensions of burnout. It is worth noting that surface acting had a positive impact on burnout, while deep acting had a negative impact on it. These findings implied that not all forms of emotional labor lead to the negative effect of increasing burnout. Some kinds of emotional labor—deep acting, in our case—contribute to reducing burnout. Similarly, Brotheridge and Grandey [1] reported that surface acting was related to feeling exhausted and detached, while deeper emotional work was related positively to personal accomplishment. As evident from the standardized coefficients, among the three dimensions of emotional labor, surface acting was the strongest predictor of burnout, followed by deep acting and intensity/variety, respectively. 

Model 3 shows how the three variables of job demands influenced burnout. First, those experiencing a higher level of role ambiguity were more likely to experience burnout than were those with less ambiguity. This confirmed the findings of a meta-research conducted by Lee and Ashforth [23] and Alarcon [24], which reported the negative impact of role ambiguity on burnout. 

Second, service workers who have heavier workloads tend to experience more burnout. In a meta-research, Alarcon [24] found that workload had a higher average weighted correlation coefficient for its relationship with emotional exhaustion than with role ambiguity. However, our study revealed a stronger correlation with ambiguity than with workload. 

Third, more customer contact had a positive impact on burnout. This confirms Scanlan and Still’s [39] findings, which revealed that customer contact influences employees’ poor wellbeing; more customer contact may increase work burden and expose the employee to the possibility of experiencing more negative events. 

Model 4 shows the effect of job resources on burnout. Two out of the three job resource variables had a negative impact on burnout. Self-efficacy significantly decreased burnout. This finding is consistent with those reported by Shoji et al. [44], Schwarzer and Hallum [45], and Jeung et al. [46]. Moreover, social support was negatively correlated with burnout, suggesting that it may help reduce burnout. Jackson et al. [52] and Demerouti et al. [22] reported that the social support in an organization has a negative impact on burnout. Both self-efficacy and social support had a similar explanatory power. This suggests that, since the self-efficacy of service workers relates only to oneself, while social support comes from peers or supervisors, not only service workers’ factors, but also those related to others in the workplace, play an important role in reducing burnout.

However, job autonomy did not influence burnout, which implies that the job design may have little impact on reducing burnout. This finding was in contrast to the results of a meta-research conducted by Alarcon [24], which found that autonomy had relationship with emotional exhaustion, cynicism, and personal accomplishment. 

Model 5, the full model, shows how all the predictors influenced burnout. As in Model 1, gender, position in the organization, and income had a positive impact on burnout. Additionally, all three job demand variables increased burnout. However, only two of the three job resource variables had a significant impact on burnout. 

The standardized coefficients revealed that among the fifteen predictors, customer contact was the strongest predictor of burnout, followed by surface acting, self-efficacy, role ambiguity, and workload, respectively. Additionally, social support and gender had some impact on burnout. This finding suggests which variables should be considered first while managing burnout. Specifically, organizational or job design should focus on identifying ways to reduce the burden caused by customer contact. On the other hand, deep acting had a weak explanatory power, followed by deep acting and position in the organization, respectively. 

Subsequently, to identify the relative importance of the four factors in explaining burnout, a linear regression was conducted, in which sociodemographic variables, emotional labor, job demands, and job resources were entered separately in Model 1 through 4, respectively. These four models explained 5.6%, 16.8%, 24.1%, and 10.5% of the variance in burnout, respectively. Thus, job demands accounted for the largest proportion of variance in burnout, followed by emotional labor, job resources, and sociodemographic factors, respectively.

Gender had a positive impact on burnout; females experienced more burnout than males did. Previous research has demonstrated that females experience more burnout than males [70], which is in contrast with the finding of Brotheridge and Grandey [1], which suggested that females did not show more burnout than males. Why do females experience more burnout? Maslach and Jackson [71] explained that, because of gender-role socialization, women emphasize caring, nurturing, and showing concern for others. Such tendencies lead females to expend more emotional effort, which finally leads to a higher level of burnout. 

### 4.3. Interaction Structure 

This study examined how JD–Rs moderate the relationship between emotional labor and burnout. To do so, we input the interaction terms (i.e., each of the three dimensions of emotional labor was multiplied by each of the six JD–R variables) into the existing regression model (Model 5) in Table 3. We used the moderation method and the procedure suggested by Baron and Kenny [72]. 

**Figure 4:**
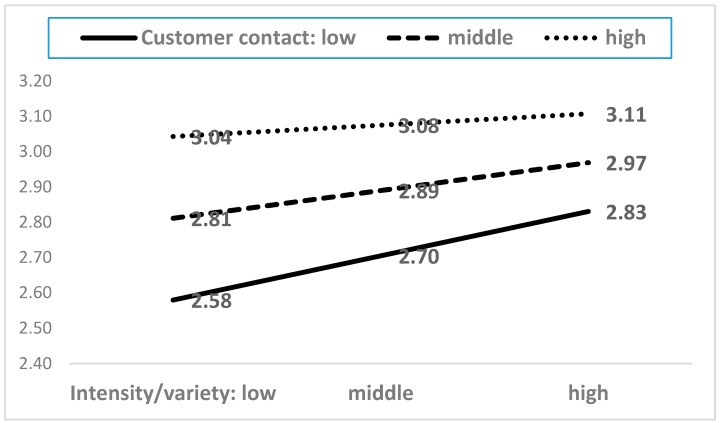
Intensity/variety (IV) × Customer contact (M) = Burnout (DV). Note: IV (Independent Variable), M (Moderator), DV (Dependent Variable).

**Figure 5:**
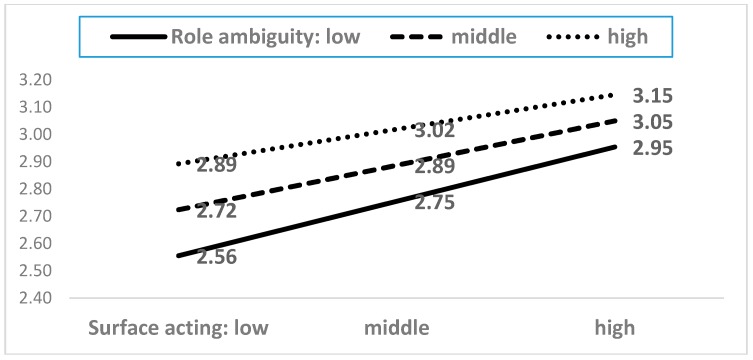
Surface acting (IV) × Role ambiguity (M) = Burnout (DV). Note: IV (Independent Variable), M (Moderator), DV (Dependent Variable).

**Figure 6:**
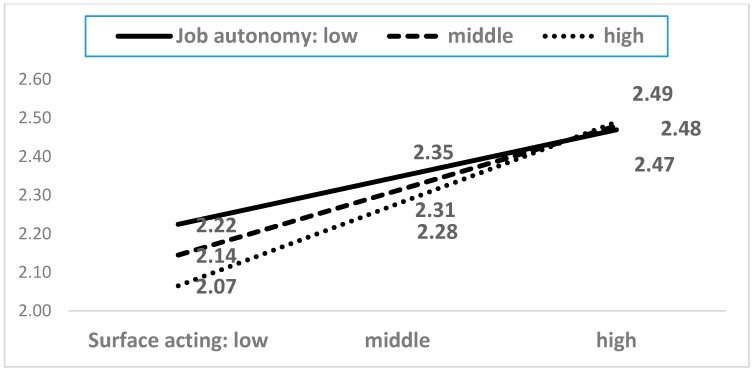
Surface acting (IV) × Job autonomy (M) = Burnout (DV). Note: IV (Independent Variable), M (Moderator), DV (Dependent Variable).

**Figure 7:**
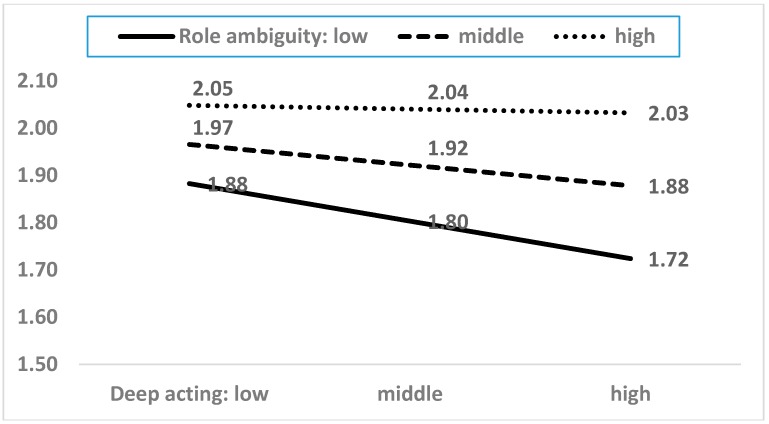
Deep acting (IV) × Role ambiguity (M) = Burnout (DV). Note: IV (Independent Variable), M (Moderator), DV (Dependent Variable).

**Figure 8:**
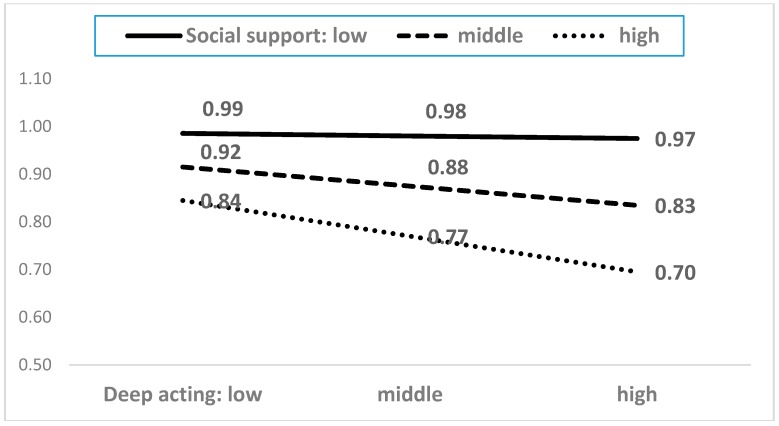
Deep acting (IV) × Social support (M) = Burnout (DV). Note: IV (Independent Variable), M (Moderator), DV (Dependent Variable).

As shown in Table A2, among the twenty-one interaction terms, five moderating terms showed statistical significance. The five moderating effects are presented in Figure 4, Figure 5, Figure 6, Figure 7 and Figure 8. In the five figures, the X-axis represents each dimension of emotional labor, while the Y-axis represents burnout. 

As evident from Figure 4, the intensity/variety dimension of emotional labor tended to increase burnout. This effect was intensified by an increase in customer contacts. This finding implies that emotional labor is more serious in employees who have had bad experiences with customers.

Figure 5 demonstrates that role ambiguity moderated the relationship between surface acting and burnout. Generally, the higher the surface acting expressed by service workers, the higher is the level of burnout they experience. This effect was more predominant in the case of greater role ambiguity. Specifically, greater ambiguity enhances the negative effect of emotional labor on burnout. 

Figure 6 shows job autonomy’s moderating effect on burnout. Bakker et al. [27] pointed out that autonomy acts as a buffer for the negative effects of emotion demand. When service workers engage in more deep acting, they generally experience a higher level of burnout. The impact of emotional labor on burnout depends on job autonomy. Specifically, surface acting would lead to a low level of burnout in the presence of low job autonomy. Thus, autonomy may help service workers cope with emotional labor because, with higher autonomy, they would be able to decide for themselves, which would attenuate emotional burden. However, this moderating effect of job autonomy becomes weaker with the increase in surface acting. These results show that the buffering effect of job autonomy may not work in situations involving higher emotional labor. 

Figure 7 shows the moderating effect of role ambiguity, which moderates the association between deep acting and burnout. The impact of deep acting on burnout depends on role ambiguity. Specifically, deep acting decreases burnout when the level of ambiguity is low. On the other hand, when role ambiguity is high, service workers experience high levels of burnout, regardless of the intensity of emotional labor. High role ambiguity constrains the impact of deep acting on burnout. These findings imply that better and clearer goal setting would reduce the negative effect of emotional labor on burnout. 

Figure 8 demonstrates the moderating role of social support in the relationship between deep acting and burnout. Employees who receive high social support would experience a lower level of burnout despite an increase in deep acting. However, in the case of low social support, deep acting would generally lead to high burnout. These results suggest that social support weakens the negative effects of emotional labor on burnout. Good relationships with supervisors and peers tend to buffer the negative impact of emotional labor. This finding confirms those of Bakker et al. [27], who reported that social support buffers the negative effects of emotional demands. 

In short, the above findings demonstrated that JD–Rs play not only a direct but also an indirect role in the effect of emotional labor on burnout. An analysis of their moderating effects revealed the negative roles of customer contact and role ambiguity, and the positive role of job autonomy and social support.

**Figure 9:**
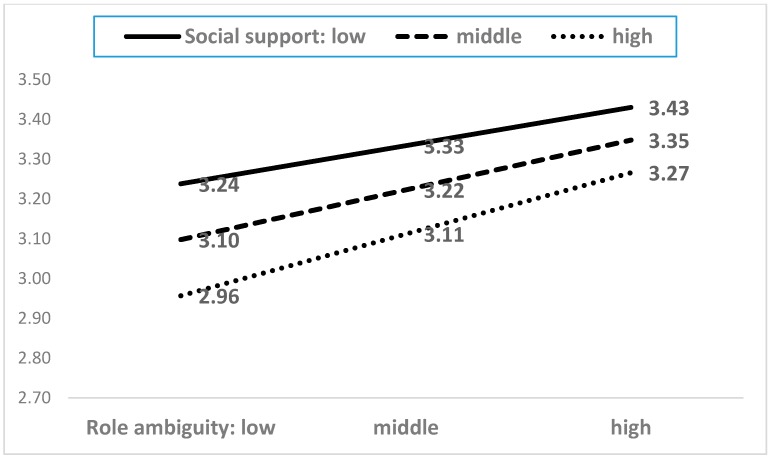
Role ambiguity (IV) × Social support (M) = Burnout (DV). Note: IV (Independent Variable), M (Moderator), DV (Dependent Variable).

Subsequently, we analyzed whether job resources moderate the impact of job demands on burnout. Of the nine possible interaction terms, only the significant interactions are presented in Figure 9. Evidently, role ambiguity increased burnout, but this effect depended on social support. Specifically, higher the level of social support, lower was the level of burnout. Thus, social support acts as a buffer for the negative effects of role ambiguity on burnout.

## 5. Conclusions

### 5.1. Findings & Summary

In Table 5, Of the fourteen hypotheses tested in the present study, twelve were supported by the data, while two were rejected. Emotional labor was found to have a systematic impact on burnout. Specifically, intensity/variety and surface acting exacerbated burnout (H1), while surface acting mitigated it (H2). Job demands and resources not only had a direct impact on burnout but also an indirect one. Among the dimensions of job demands, role ambiguity (H3-1), workload (H4-1), and customer contact (H5-1) had a negative impact on burnout, while self-efficacy (H6-1) and social support (H8-1) had a positive impact on it. Among the JD–Rs, job autonomy did not have a significant impact on burnout (H7-1); however, the JD–Rs moderated the relationship between emotional labor and burnout. Among the twenty-four interaction terms, five were statistically significant. In case of ambiguity, one out of three interaction terms show significance. Role ambiguity moderated the relationship between surface acting, deep acting, and burnout (H3-2). Customer contact (5-2) moderated the impact of intensity/variety on burnout. On the other hand, job autonomy (H7-1) and social support (H8-1) moderated the impact of both surface and deep acting. Among the dimensions of JD–R, social support moderated the relationship between job ambiguity and burnout (H9).

### 5.2. Discussion

Our study analyzed how emotional labor influences burnout, and examined whether JD–Rs have a direct and indirect impact on burnout. Findings revealed that JD–Rs were not only the main factor in influencing burnout but also the major moderators in the relationship between emotional labor and burnout. Our results clearly showed the role of emotional labor and JD–R in burnout. Our main findings were as follows. 

A simple means analysis showed that women experience a higher level of burnout than men do. Additionally, the degree of burnout declined with an increase in age. Burnout was high in individuals in the middle level in an organization. Burnout was lower when income was higher. Further, university graduates had a higher level of burnout than high school graduates did. A regression analysis showed the significant impact of gender, position in the organization, and income on burnout. A previous study confirmed that females experience more burnout than males do [69]. A higher position in an organization and higher income level may serve as resources to prevent burnout. 

The regression results revealed the significant impact of emotional labor and JD–Rs on burnout. Specifically, the three dimensions of emotional labor have contrasting effects on burnout. Intensity/variety and surface emotional labor had positive effects on burnout, while deep acting had a negative effect. Evidently, deep acting reduces exhaustion, which confirms Hochschild’s argument that surface acting produces negative outcomes, while deep acting induces positive results [2]. Among the three dimensions of emotional labor, surface acting explained most of the variance in burnout, followed by intensity/variety and deep acting, respectively. This confirmed the findings reported by Brotheridge and Grandey [1], which suggested that surface acting is strongly associated with emotional exhaustion.

Among the dimensions of job demands, role ambiguity, job overload, and customer contact had a positive impact on burnout. Among these three variables, customer contact had the highest explanatory power, followed by role ambiguity and workload, respectively. This finding seems logical, in that service workers’ main task is to deal with customers. On the other hand, among the three dimensions of job resources, self-efficacy and social support affected burnout significantly. Specifically, the level of burnout decreased with an increase in self-efficacy and social support. However, as compared to social support, self-efficacy had a higher explanatory power.

Based on our findings related to the impact of JD–Rs, we conclude that job demands have a negative impact on burnout, while job resources have a positive effect on it. This finding confirms the basic argument about the contrasting roles of job demands and resources [13,73].

Among all the fifteen independent variables, customer contact had the highest explanatory power, followed by surface acting, self-efficacy, role ambiguity, and workload, respectively. Additionally, social support and gender had an impact on burnout to some extent. 

The present study also found that JD–Rs moderate the relationship between emotional labor and burnout. Intensity/variety and surface acting tended to increase burnout; however, such effects depend on customer contact and role ambiguity. The tendency of emotional labor to increase burnout was exacerbated by experiences with difficult customers and high ambiguity. In case of job autonomy, when emotional labor was low, low autonomy was more likely to lead to high burnout than high autonomy was. However, when emotional labor was high, burnout was high regardless of the level of autonomy. Moreover, in the presence of high role ambiguity, deep acting was more likely to lead to high burnout, while low ambiguity weakened the effect of emotional labor on increasing burnout. Finally, high social support mitigated the negative effects of emotional labor on burnout. However, when social support was low, emotional labor was directly linked to burnout.

In short, our findings provide a theoretical understanding of emotion management, JD–Rs, and burnout. First, we provided confirmatory findings on emotional labor. Emotional labor significantly affects burnout. This study showed that the emotional labor of surface acting had a decisive influence on burnout. Second, job resources and job demands perform contrasting functions in burnout; the former reduces burnout, while the latter increases it. Particularly, customer contact, role ambiguity, and workload had a significant positive effect on burnout, while self-efficacy and social support had a negative impact on it. Finally, job demands and resources play the role of moderating variables. Specifically, role ambiguity and customer contact increased burnout, while job autonomy and social support decreased it. 

### 5.3. Theoretical Implications 

The present analysis demonstrated that both emotional labor and JD–Rs explain the large variance in burnout. Our study makes three major theoretical contributions. First, it contributes to the literature on emotional labor and burnout by revealing the mechanism of their connection. Though several studies have suggested that emotional labor leads to burnout, few studies have clarified this mechanism. Further, previous studies did not confirm the moderating role of JD–Rs. For example, based on a meta-review, Hülsheger and Schewe [7] concluded that the type of service interaction, one of the job demands, did not emerge as a moderator. However, they attributed this finding to the small sample size in primary studies and the lack of clear distinctions in the differences between service encounters and relationships based on occupation or job title. The present study confirmed the moderating effect of customer contact [42], role ambiguity [7], autonomy [54], and social support [4]. Moreover, it revealed the interaction effect of JD–Rs. The JD–R model predicts that job resources decrease the negative effect of job demands on exhaustion [57]. Bakker et al. [53] reported that the effect of job resources on cynicism was particularly strong when employees faced many job demands. The present study found that social support moderates the relationship between role ambiguity and burnout. 

Second, previous studies reported that deep acting is better than surface acting because the latter has more detrimental effects on work-related outcomes. For instance, according to Hülsheger and Schewe [7], deep acting had a weak relationship with impaired wellbeing and job attitudes, but a positive relationship with emotional performance and customer satisfaction. Springer and Oleksa [74] reported that surface acting leads to an increase in burnout. However, recently, Hülsheger and Schewe [7] argued that emotional labor does not necessarily harm employees. Moreover, Grandey and Melloy [75] pointed out that emotional regulation led to the good–bad dichotomy of deep and surface acting. 

The new model proposed in the present study shows that surface and deep acting are not always beneficial or harmful, because their paths are moderated by individual and contextual factors. Specifically, we found that with an increase in contact with customers, surface acting increases exhaustion. However, these negative effects are reduced when there is less customer contact, less role ambiguity, and high autonomy. Additionally, deep acting reduces burnout. However, this mitigating effect decreases when role ambiguity is high and when social support is low. 

Third, Schaufeli and Taris [57] suggested that the JD–R model could include an extremely wide set of job and personal characteristics and outcomes. They stressed that “the model can be tailored to the specific needs of an organization, given any specific situation” (p. 59). This implies that the validity of the JD–R model depends on the given organization. The present study confirmed the validity of the JD–R model in service organizations. At the variable level, our study showed that contact with customers in service organizations had the greatest impact on burnout. In addition, customer contact moderated the effect of emotional labor on exhaustion. This direct and indirect impact of customer contact reflects the unique characteristic of service organizations.

Fourth, there is controversy over the direction of causality in the relationship between JD–Rs and emotional labor. Our findings show that emotional labor determines JD–Rs. However, other studies showed that JD–Rs affect emotional labor. For example, Wang et al. [76] reported that JD–Rs predict emotional labor. They suggested that negative display rules; high levels of job demands; frequent contacts with customers; and lack of autonomy and social support are significantly related to surface acting; while display rules; opportunities to display various emotions; and frequent, intensive, and long periods of contact with customers are significantly related to deep acting. The present study demonstrated that JD–Rs mainly moderate the impact of emotional labor on burnout. 

### 5.4. Practical Implications

The present findings call for more practical and active emotional management at the individuals and organizational levels. In particular, we collected data from local government, it gives more practical implication for labor policy at local context. Managerial efforts are needed to reduce the emotional labor of frontline workers. According to Humphrey et al. [54], a first step to manage emotional labor is to identify employees with a higher tendency to engage in surface rather than deep acting, and specific situations that trigger the use of surface acting. Appropriate solutions could then be sought. Jeung et al. [46] reported that both stress management programs and personal coping skills are important for reducing the adverse outcomes of emotional labor. This implies that multilevel approaches across individual and organizational levels are essential for managing emotional labor. 

First, to decrease emotional labor at the individual level, it is necessary to enhance workers’ abilities and competencies, and to encourage a positive personality through behavior modification. The present study showed that surface acting has a significant effect on burnout. These results emphasize the need to manage surface acting. One way of doing so is to regularize emotional expression. Springer and Oleksa [74] suggested that organizations develop standards for the expression of emotions and preventive actions, such as identification with the organization. Moreover, emotion regulation has been discussed as a method to manage emotional labor. Gross [77] defined emotion regulation as the ability to manage emotional experiences and expressions. Zhao et al. [78] demonstrated that emotion regulation played a significant role in reducing burnout. Therefore, the need to design programs that help employees enhance their emotion regulation ability is acknowledged. Gross [79] proposed specific emotion regulation strategies, including attentional deployment, cognitive reappraisal and suppression. Grandey [9] suggested that reducing deep acting by modifying feelings through attentional deployment and cognitive change, and managing surface acting by modifying expressions through response modulation, could be effective emotion regulation strategies. Similarly, Andela and Truchot [80] demonstrated that cognitive change is associated with low levels of burnout, while attentional deployment is positively related to burnout. However, it is risky to teach employees to regulate their emotions because they are often merely viewed as a commodity.

Second, at the organizational level, the need to introduce stress management programs to reduce the adverse outcomes of emotional labor, as well as to improve the coping repertoire of employees to strengthen their personal potential to suit organizational goals, has been expressed [46]. All of management is intentional. Gross [79] proposed situational selection and modification as specific emotion regulation strategies. The two options depend not only on individual but also on organizational level efforts. Jeung et al. [46] suggested that enhancing employees’ abilities and competencies, and encouraging a positive personality through behavior modification, are also necessary interventions that need to be implemented at the organizational level.

Moreover, as a part of human resource management, selecting, rewarding, developing, and evaluating individuals with competencies essential for performing emotional labor could help service organizations improve their human capital in the long run [81]. When selecting employees and teams, organizations should consider a positive emotional attitude. Moreover, evaluations, rewards, and compensation systems should be designed to induce the desired emotional behavior. Positive emotional behavior should be rewarded and compensated appropriately. Training programs should be oriented to increase emotional intelligence and healthy emotional expression in employees [82]. Organizations should try to develop and implement practical interventions to train service workers on ways to manage their emotions. Gross [79] suggested that it is important to encourage emotional labor to use reappraisal of their mind. 

Further, the company’s health policies, rewards for service, and formal support groups could help create a workplace climate that protects employees from socioemotional work stressors [83,84]. Organizations need to create a positive and friendly emotional climate in which managers should stress on healthy emotional expression. The model refers to attention to emotion perception in the workplace anxieties; warm and sincere expressions of positive emotion; and constructively assertive and appropriate expressions of negative emotion [82]. 

Next, besides emotional labor, burnout itself is a target for management. Maslach and Goldberg [8] suggested two approaches for preventing burnout, which focus on the interaction between personal and situational factors. First, one needs to increase workers’ engagement with their work by creating a better “fit” between the individual and the job. Second, it is important to reframe the response to burnout by modifying the individual’s response to the job setting, in terms of perceptions and decisions about its risk factors for burnout, and by reframing the causal antecedents of burnout within the six areas of mismatch between the person and the job (p. 69). Moreover, Bakker et al. [13] stressed on the need to increase job resources to reduce burnout. Particularly, social support and performance feedback can be optimized by good job design and training. Grandey et al. [85] stressed on the importance of the organizational culture. They demonstrated that burnout can be reduced by the presence of a “climate of authenticity,” which is the perceived acceptance of and respect for unit members’ expression of emotions they experience when interacting with coworkers. It replenishes resources and buffers the strain caused by emotional labor.

Our analysis confirmed that JD–Rs significantly influence burnout. Particularly, job demands are more important than job resources. Therefore, a key to developing appropriate interventions for burnout is the recognition of emotional labor as being an “affective job demand” [81]. This reflects the unique characteristic of service organizations. Specifically, in service organizations, employers are constantly in contact with customers, which causes emotional labor to increase burnout. Our findings showed that contact with customers explained most of the variance in burnout, followed by self-efficacy, role ambiguity, workload, and social support, respectively.

### 5.5. Limitations

While our analysis showed the direct and indirect effects of JD–Rs on burnout, it has limitations. First, there are limitations to adopting a few predictor variables. Lee and Ashforth [23] found that not only role ambiguity and workload, but also role conflicts, role stress, stressful events, and work pressure increase burnout. However, our study included only two of these six variables. Future studies should examine the role of other significant variables.

Second, although burnout comprises three variables—emotional exhaustion, depersonalization, and reduced personal accomplishment—at the theoretical level [86]. This study used these variables without dividing them into these three dimensions because of the poor reliability of the measurement instrument used. In future research, the need to increase the reliability of the measurement tool is acknowledged.

Third, in previous studies, the intensity/variety of emotional labor was considered to comprise different dimensions. However, the present study considered it only as one dimension owing to the lack of validity and reliability of the measurement instrument used.

Fourth, there can be various relationships between the dimensions of JD–Rs and one of the dimensions of emotional labor, which could yield individual hypotheses on their moderating effects. However, because of a weak theoretical and logical basis, this study failed to propose hypotheses on these relationships. 

Fifth, the present study utilized data from four service groups. However, it did not aim to test the effect of occupational differences on the study variables. Brotheridge and Grandey [1] reported that the levels of burnout and emotional labor differed across occupations. Future research should compare different occupational groups. Moreover, it is necessary to increase the representativeness of the sample and the generalizability of the present results by covering more diverse groups.

Sixth, Diefendorff et al. [20] reported that unit-level display rules relate to burnout indirectly through individual-level display rule perceptions and emotion regulation strategies. Moreover, unit-level display rules also interacted with individual-level dispositional affectivity to predict employees’ use of emotion regulation strategies. They well demonstrated the role of unit-level display rules and dispositional affectivity; however, the present study did not examine the same.

## Figures and Tables

**Table 1:** Measurement and reliability.

Concept	Measures	Factor Loading			Reliability
		1	2	3	
1. Intensity/variety	I often have to express my inner feelings heartily during work.	0.816	0.037	0.212	0.894
	I often have to express my inner feelings very strongly.	0.877	0.076	0.084	
	I have to express many kinds of emotions in my job.	0.888	0.146	0.084	
	I have to express very different emotions when meeting with customers.	0.838	0.137	0.096	
2. Surface acting	I often have to suppress my frank feelings when I deal with customers.	0.196	0.532	0.143	0.787
	When I deal with customers, I pretend to have feelings that are not in my heart.	0.123	0.807	−0.047	
	Regardless of what is on my mind, I make a good facial expression.	−0.009	0.846	0.264	
	I pretend to be good when I treat customers.	0.044	0.839	0.251	
3. Deep acting	I try to feel the emotions that I have to show when I treat customers.	0.239	0.311	0.654	0.759
	I try to feel the emotions that I have to show in my work.	0.234	0.129	0.749	
	I try to show my clients good feelings.	0.018	0.216	0.757	
	I try to show my sincere emotions to customers to create a good organizational image.	0.047	0.01	0.838	
4. Role ambiguity	I do not have enough policies or guidelines to refer to when I do my work. I am working under very vague instructions or regulations.				0.715
5. Workload	There is not enough time to deal with tasks that must be done in the office. I work hard for a long time when I work.				0.683
6. (Negative) Customer contact	Often the demands of customers are excessive. Customers sometimes attack with words. Customers get angry with us for very minor problems. Customers often ask for impossible or contradictory things.				0.858
7. Self-efficacy	I have a strong sense of pride in my work knowledge and abilities. I am confident that I am able to work better than most of my colleagues at work.				0.773
8. Job autonomy	I have the autonomy and discretion to decide what services to offer. I have a fair amount of autonomy in what I do in my work.				0.783
9. Social support	I can depend on my supervisors when my job gets more difficult. My supervisors will help me in dealing with difficult situations. I can depend on my colleagues when my job is difficult. My colleagues help me deal with difficult situations.				0.854
10. Burnout	I am emotionally exhausted by my work. I feel exhausted after a day’s work. I have less affection for and interest in my current job than I used to. I am skeptical about how my current job will benefit me.				0.853

Note: A factor analysis was conducted using the principle components factor method with varimax rotation.

**Table 2:** The average variance extracted, composite reliability, and correlations for the models tested.

Four-Factor Model				Three-Factor Model			
Latent Var.	AVE	CR	Correlation (Correlation Squared)	Latent Var.	AVE	CR	Correlation (Correlation Squared)
Intensity (A)	0.713	0.832	A&B = 0.887 *** (0.787) A&C = 0.183 *** (0.033) A&D = 0.380 *** (0.144) B&C = 0.243 *** (0.059) B&D = 0.357 *** (0.127) C&D = 0.514 *** (0.264)	Intensity/variety (A)	0.681	0.895	A&B = 0.228 *** (0.052) A&C = 0.386 *** (0.149) B&C = 0.513 *** (0.263)
Variety (B)	0.761	0.864					
Surface Acting (C)	0.524	0.805		Surface Acting (B)	0.524	0.805	
Deep Acting (D)	0.484	0.786		Deep Acting (C)	0.483	0.786	

Note: AVE (Average Variance Extracted), CR (Composite reliability); * *p* < 0.05; ** *p* < 0.01; *** *p* < 0.001.

**Table 3:** Pearson’s correlations and partial correlations among study variables.

		1	2	3	4	5	6	7	8	9	10
Emotional labor	1. Intensity/variety		0.262 ***	0.311 ***	0.203 ***	0.227 ***	0.123 ***	0.159 ***	0.236 ***	0.007	0.174 ***
	2. Surface acting	0.247 ***		0.393 ***	0.263 ***	0.212 ***	0.391 ***	0.051 *	0.052 *	−0.030	0.363 ***
	3. Deep acting	0.314 ***	0.390 ***		0.061 **	0.188 ***	0.161 ***	0.345 ***	0.264 ***	0.246 ***	0.020
Job demands	4. Role ambiguity	0.194 ***	0.260 ***	0.055 **		0.274 ***	0.238 ***	−0.091 **	−0.153 ***	−0.248 ***	0.379 ***
	5. Workload	0.223 ***	0.209 ***	0.193 ***	0.266 ***		0.234 ***	0.140 ***	0.081 ***	0.046 *	0.267 ***
	6. Customer contact	0.113 ***	0.379 ***	0.153 ***	0.235 ***	0.222 ***		0.095 ***	−0.042	−0.064 **	0.384 ***
Job resources	7. Self-efficacy	0.166 ***	0.009	0.335 ***	−0.108 ***	0.130 ***	0.072 ***		0.329 ***	0.302 ***	−0.211 ***
	8. Job autonomy	0.235 ***	0.021	0.255 ***	−0.154 ***	0.083 ***	−0.052 **	0.346 ***		0.371 ***	−0.161 ***
	9. Social support	−0.008	−0.026	0.233 ***	−0.231 ***	0.047 *	−0.067 ***	0.262 ***	0.353 ***		−0.271 ***
	10. Burnout	0.153 ***	0.375 ***	0.022	0.372 ***	0.267 ***	0.373 ***	−0.256 ***	−0.177 ***	−0.256 ***	
Mean		2.930	3.330	3.440	3.056	3.296	3.730	3.561	3.140	3.620	3.229
SD		0.804	0.703	0.590	0.797	0.738	0.690	0.747	0.806	0.710	0.831

Note: * *p* < 0.05; ** *p* < 0.01; *** *p* < 0.001; SD = Standard Deviation.

**Table 4:** Findings of the multiple regression analysis on burnout.

		Model 1			Model 2			Model 3			Model 4			Model 5		
		B	SE	Beta	B	SE	Beta	B	SE	Beta	B	SE	Beta	B	SE	Beta
Constant		3.050	0.214		2.112	0.130		6222	0.125		4.931	0.130		1.634	0.250	
Sociodemographic Factor	Gender	0.291 ***	0.048	0.173										0.186 ***	0.039	0.110
	Age	−0.002	0.006	−0.022										0.002	0.005	0.018
	Year of employment	−0.004	0.006	−0.043										−0.004	0.005	−0.041
	Position in the organization	0.204 ***	0.057	0.121										0.140 ***	0.046	0.083
	Household income	−0.117 **	0.054	−0.065										−0.088 **	0.044	−0.049
	Education Level	−0.041	0.045	−0.024										−0.056	0.036	−0.033
Emotional labor	Intensity/variety				0.108 ***	0.026	0.104							0.083 ***	0.024	0.080
	Surface acting				0.492 ***	0.030	0.416							0.239 ***	0.030	0.201
	Deep acting				−0.243 ***	0.037	−0.173							−0.073 **	0.035	−0.052
Job demand	Role ambiguity							0.283 ***	0.025	0.272				0.161 ***	0.024	0.155
	Workload							0.148 ***	0.027	0.132				0.168 ***	0.026	0.149
	Customer contact							0.336 ***	0.028	0.280				0.274 ***	0.028	0.228
Job resource	Self-efficacy										−0.212 ***	0.029	−0.191	−0.216 ***	0.027	−0.193
	Job autonomy										−0.045	0.028	−0.044	−0.039	0.025	−0.038
	Social support										−0.222 ***	0.031	−0.190	−0.150 ***	0.028	−0.128
F-value		14.125 ***			101.813 ***			159.112 ***			59.265 ***			60.656 ***		
R^2^/Adjusted R^2^		0.056/0.052			0.168/0.166			0.241/0.239			0.105/0.103			0.391/0.385		

Note: * *p* < 0.05; ** *p* < 0.01; *** *p* < 0.001.

**Table 5:** Summary of the hypothesis testing.

Hypothesis	B	*p*-Value	Accept/Reject
H1: Intensity/variety (+)	0.083	0.001	**Accept**
H2: Surface acting (+)/Deep acting (−)	0.239/−0.073	0.000/0.040	**Accept**
H3-1: Role ambiguity (+)	0.161	0.000	**Accept**
H3-2: Role ambiguity as moderator	−0.065/0.076 *	0.016/0.018	**Partially accept**
H4-1: Workload (+)	0.168	0.000	**Accept**
H4-2: Workload as moderator	-	a	Reject
H5-1: Customer contact (+)	0.274	0.000	**Accept**
H5-2: Customer contact as moderator	−0.084	0.001	**Partially accept**
H6-1: Self-efficacy (−)	−0.216	0.000	**Accept**
H6-2: Self-efficacy as moderator	-	N.S.	Reject
H7-1: Job autonomy (−)	−0.039	0.117	Reject
H7-2: Job autonomy as moderator	0.079	0.003	**Partially accept**
H8-1: Social support (−)	−0.150	0.000	**Accept**
H8-2: Social support as moderator	−0.035	0.016	**Partially accept**
H9: Job resource moderator for job demand (IV)	0.051	0.043	**Partially accept**

Note: 1. (+); positive impact, (−); negative impact. 2. Burnout was the dependent variable in all hypotheses. 3. (*) Independent variables were surface acting/deep acting. 4. (IV): Independent Variable. 5. N.S.: Not Significance.

## Appendix A

**Table A1:** Population, Sample and Sampling Method.

Category	Population	Sampling Method	No. of Respondents	Response Rate	Final No. of Cases for Analysis
Fire-fighters	Fire-fighters in Gyeonggi-do Firefighting (No. of employers = 7071).	23 fire stations were randomly selected from 34 fire ones (excluding 11 in the northern ones), then sample quota was assigned to each fire station. Next, the number of questionnaires was assigned to each department in proportion to the number of employers in each department. Next, it selects randomly respondents from each department. Finally, after asking the respondents to participate on voluntary bas, survey was distributed.	510	90.2%	433
Police	Police officers un Gyeonggi Provincial Police Agency (No. of employers = 13,819)	22 police stations were randomly selected from 30 ones in Gyeonggi, and then allocated a quoted number of samples to each police station. Then, the number of the questionnaires has been allocated in proportion to the number of workers in civil service department in each police station. Finally, randomly extracted randomly extracted the subjects of the final questionnaire response in each department.	430	82.1%	342
General civil servants	Civil servants in Suwon city (No. of employers = 2487)	According to the assigned quota, departments were extracted from civil affairs department the in headquarters, the ward office civil affairs department in districts, and the local civil offices, all of which belonged to Suwon city. We allocated the number of questionnaires in proportion to the number of employers in each department. The final respondents to the questionnaire were randomly extracted in each department.	430	91.2%	375
Nurse	Nurses in the Gyeonggi Provincial Medical Center. (No. of employers = 572)	According to the hospital size (large, middle, small), the number of samples was allocated to six public hospitals belonging to the Gyeonggi Provincial Medical Center. Then, we randomly extracted the specific department from the list of nursing related departments in each hospital. Then, the number of questionnaires was allocated in proportion to the number of nursing staff working in the extracted department. The final target for the questionnaire were randomly extracted in each department.	430	89.5%	367
Sum	-	-	**1800**	**88.3%**	**1517**

**Table A2:** Analysis of Interaction Effect and Simple Slope Test.

**Intensity/variety (IV) × Customer contact (M) = Burnout (DV)**							**Surface acting (IV) × Role ambiguity (M) = Burnout (DV)**						
	B	SE	beta	B	SE	beta		B	SE	beta	B	SE	beta
Intensity/variety	0.083 **	0.024	0.08	0.098 ***	0.025	0.095	Surface acting	0.239 ***	0.03	0.201	0.232 ***	0.03	0.195
Customer contact	0.274 ***	0.028	0.228	0.268 ***	0.028	0.223	Role Ambiguity	0.161 ***	0.024	0.155	0.166 ***	0.024	0.159
Interaction Term	-			−0.084 *	0.028	−0.065	Interaction Term	-			−0.065 *	0.027	−0.051
F-value	61.860 ***			58.856 ***			F-value	61.860 ***			58.556 ***		
R² square	0.396			0.4			R² square	0.396			0.399		
R² square Change	0.39			0.393			R² square Change	0.39			0.92		
Simple Slope Test	Law	B = 0.160 *** se = 0.034 t = 4.524					Simple Slope Test	Law	B = 0.284 *** se = 0.035 t = 8.070				
	Middle	B = 0.098 *** se = 0.024 t = 4.065						Middle	B = 0.232 *** se = 0.032 t = 7.305				
	High	B = 0.040 se = 0.028 t = 1.420						High	B = 0.180 *** se = 0.039 t = 4.661				
Effect Size	0.003						Effect Size	0.003					
**Surface acting (IV) × Job autonomy (M) = Burnout (DV)**							**Deep acting (IV) × Role ambiguity (M) = Burnout (DV)**						
	B	SE	beta	B	SE	beta		B	SE	beta	B	SE	beta
Surface Action	0.239 ***	0.03	0.201	0.238 ***	0.03	0.201	Deep acting	−0.073 *	0.035	−0.052	−0.074 *	0.035	−0.053
Job Autonomy	−0.039	0.025	−0.038	−0.043	0.025	−0.042	Role Ambiguity	0.161 ***	0.024	0.155	0.149 ***	0.025	0.143
Interaction Term	-			0.079 *	0.027	0.062	Interaction Term	-			0.076 *	0.032	0.051
F-value	61.860 ***			58.843 ***			F-value	61.860 ***			58.538 ***		
R² square	0.396			0.4			R² square	0.396			0.399		
R² square Change	0.39			0.393			R² square Change	0.39			0.392		
Simple Slope Test	Law	B = 0.174 *** se=0.037 t = 4.700					Simple Slope Test	Law	B = −0.135 ** se = 0.044 t = −3.079				
	Middle	B = 0.238 *** se=0.030 t = 7.875						Middle	B = −0.074 * se = 0.036 t = −2.080				
	High	B = 0.302 *** se=0.037 t = 8.184						High	B = −0.013 se = 0.043 t = −0.312				
Effect Size	0.003						Effect Size	0.003					
**Deep acting (IV) × Social support (M) = Burnout (DV)**							**Customer contact (IV) × Role ambiguity (M) = Burnout (DV)**						
	B	SE	beta	B	SE	beta		B	SE	beta	B	SE	beta
Deep acting	−0.073 *	0.035	−0.052	−0.068	0.035	−0.049	Customer contact	0.274 ***	0.028	0.228	0.264 ***	0.028	0.220
Social Support	−0.150 ***	0.028	−0.128	−0.148 ***	0.028	−0.126	Role Ambiguity	0.161 ***	0.024	0.155	0.172 ***	0.025	0.166
Interaction Term	-			−0.035 *	0.034	−0.050	Interaction Term	-			−0.085 *	0.027	−0.066
F-value	61.860 ***			58.555 ***			F-value	61.860 ***			58.966 ***		
R² square	0.396			0.399			R² square	0.396			0.401		
R² square Change	0.39			0.392			R² square Change	0.390			0.394		
Simple Slope Test	Law	B = −0.009 se = 0.044 t = −0.207					Simple Slope Test	Law	B = 0.333 *** se = 0.034 t = 9.911				
	Middle	B = −0.068 se = 0.035 t = −1.925						Middle	B = 0.265 *** se = 0.028 t = 9.456				
	High	B = −0.127 ** se=0.042 t = −3.031						High	B = 0.196 *** se = 0.037 t = 5.273				
Effect Size	0.003						Effect Size	0.004					
**Role ambiguity (IV) × Social support (M) = Burnout (DV)**													
	B	SE	beta	B	SE	beta							
Role ambiguity	0.161 ***	0.024	0.155	0.156 ***	0.024	0.150							
Social Support	−0.150 ***	0.028	−0.128	−0.156 ***	0.028	−0.133							
Interaction Term	-			0.051 *	0.025	0.043							
F-value	61.860 ***			58.377 ***									
R² square	0.396			0.398									
R² square Change	0.390			0.391									
Simple Slope Test	Law	B = 0.120 *** se = 0.032 t = 3.771											
	Middle	B = 0.156 *** se = 0.024 t = 6.372											
	High	B = 0.192 *** se = 0.029 t = 6.679											
Effect Size	0.002												

Note: * *p* < 0.1, ** *p* < 0.05, *** *p* < 0.001.

## References

[1] Brotheridge C.M., Grandey A.A. (2002). Emotional labor and burnout: Comparing two perspectives of “people work”. J. Vocat. Behav..

[2] Hochschild A.R. (1983). The Managed Heart: Commercialization of Human Feeling.

[3] Hochschild A.R. (1979). Emotion work, feeling rules, and social structure. Am. J. Sociol..

[4] Morris J.A., Feldman D.C. (1996). The dimensions, antecedents, and consequences of emotional labor. Acad. Manag. Rev..

[5] Zapf D. (2002). Emotion work and psychological well-being: A review of the literature and some conceptual considerations. Hum. Resour. Manag. Rev..

[6] Morris J.A., Feldman D.C. (1997). Managing emotions in the workplace. J. Manag. Issues.

[7] Hülsheger U.R., Schewe A.F. (2011). On the costs and benefits of emotional labor: A meta-analysis of three decades of research. J. Occup. Health Psychol..

[8] Maslach C., Goldberg J. (1998). Prevention of burnout: New perspectives. Appl. Prev. Psychol..

[9] Grandey A. (2000). Emotion regulation in the workplace: A new way to conceptualize emotional labor. J. Occup. Health Psychol..

[10] Grandey A. Emotional labor: A concept and its correlates. Proceedings of the 1st Conference on Emotions in Organizational Life.

[11] Freudenberger H.J. (1974). Staff burnout. J. Soc. Issues.

[12] Maslach C., Jackson S.E. (1981). The measurement of experienced burnout. J. Occup. Behav..

[13] Bakker A.B., Demerouti E., Sanz-Vergel A.I. (2014). Burnout and work engagement: The JD-R approach. Annu. Rev. Organ. Psychol. Organ. Behav..

[14] Schaufeli W.B., Leiter M., Maslach C., Jackson S.E. (1996). The Maslach Burnout Inventory–General Survey. MBI Manual.

[15] Maslach C. (1982). Burnout: The Cost of Caring.

[16] Zapf D., Seifert C., Schmutte B., Mertini H., Holz M. (2001). Emotion work and job stressors and their effects on burnout. Psychol. Health.

[17] Grandey A.A. (2003). When “the show must go on”: Surface acting and deep acting as determinants of emotional exhaustion and peer-rated service delivery. Acad. Manag. Rev..

[18] Kim S., Park C. (2017). Street-level bureaucrats’ job burnout and engagement: Focusing on role of job demands-resources model and CSS (customer-related social stressors). Korean Public Adm. Rev..

[19] Scott B.A., Barnes C.M. (2011). A multilevel field investigation of emotional labor, affect, work withdrawal, and gender. Acad. Manag. J..

[20] Diefendorff J.M., Erickson R.J., Grandey A.A., Dahling J.J. (2011). Emotional display rules as work unit norms: A multilevel analysis of emotional labor among nurses. J. Occup. Health Psychol..

[21] Bakker A.B., Demerouti E., De Boer E., Schaufeli W.B. (2003). Job demands and job resources as predictors of absence duration and frequency. J. Vocat. Behav..

[22] Demerouti E., Bakker A., Nachreiner F., Schaufeli W.B. (2001). The job demands–resources model of burnout. J. Appl. Psychol..

[23] Lee R.T., Ashforth B.E. (1996). A meta-analytic examination of the correlates of the three dimensions of job burnout. J. Appl. Psychol..

[24] Alarcon G.M. (2011). A meta-analysis of burnout with job demands, resources, and attitudes. J. Vocat. Behav..

[25] Christian M.S., Garza A.S., Slaughter J.E. (2011). Work engagement: A quantitative review and test of its relations with task and contextual performance. Pers. Psychol..

[26] Schaufeli W.B., Bakker A.B. (2004). Job demands, job resources, and their relationship with burnout and engagement: A multi-sample study. J. Organ. Behav..

[27] Bakker A.B., Demerouti E., Euwema M.C. (2005). Job resources buffer the impact of job demands on burnout. J. Occup. Health Psychol..

[28] Schwab R.L., Iwanicki E.F. (1982). Perceived role conflict, role ambiguity, and teacher burnout. Educ. Adm. Q..

[29] Papastylianou A., Kaila M., Polychronopoulos M. (2009). Teachers’ burnout, depression, role ambiguity and conflict. Soc. Psychol. Educ..

[30] Tunc T., Kutanis R.O. (2009). Role conflict, role ambiguity, and burnout in nurses and physicians at a university hospital in Turkey. Nurs. Health Sci..

[31] Brooking J.B., Bolton B., Brown C.E., McEvoy A. (1985). Self-reported job burnout among female human service professionals. J. Occup. Behav..

[32] Kwak H., McNeeley S., Kim S. (2018). Emotional labor, role characteristics, and police officer burnout in South Korea: The mediating effect of emotional dissonance. Police Q..

[33] Tetrick L.E., Slack K.J., Da Silva N., Sinclair R.R. (2000). A comparison of the stress–strain process for business owners and nonowners: Differences in job demands, emotional exhaustion, satisfaction, and social support. J. Occup. Health Psychol..

[34] Maslach C., Schaufeli W.B., Leiter M.P. (2001). Job Burnout. Annu. Rev. Psychol..

[35] Greenglass E.R., Burke R.J., Fiksenbaum L. (2001). Workload and burnout in nurses. J. Community Appl. Soc. Psychol..

[36] Van Bogaert P., Clarke S., Willems R., Mondelaers M. (2013). Nurse practice environment, workload, burnout, job outcomes, and quality of care in psychiatric hospitals: A structural equation model approach. J. Adv. Nurs..

[37] Park H.I., O’Rourke E., O’Brien K.E. (2014). Extending conservation of resources theory: The interaction between emotional labor and interpersonal influence. Int. J. Stress Manag..

[38] Huang J.L., Chiaburu D.S., Zhang X., Li N., Grandey A.A. (2015). Rising to the challenge: Deep acting is more beneficial when tasks are appraised as challenging. J. Appl. Psychol..

[39] Scanlan J.N., Still M. (2013). Job satisfaction, burnout and turnover intention in occupational therapists working in mental health. Aust. Occup. Ther. J..

[40] Cordes C.L., Dougherty T.W. (1993). A review and an integration of research on job burnout. Acad. Manag. Rev..

[41] Grandey A., Dickter D.N., Sin H.P. (2004). The customer is not always right: Customer aggression and emotion regulation of service employees. J. Organ. Behav..

[42] Côté S. (2005). A social interaction model of the effects of emotion regulation on work strain. Acad. Manag. Rev..

[43] Sliter M., Jex S., Wolford K., McInnerney J. (2010). How rude! emotional labor as a mediator between customer incivility and employee outcomes. J. Occup. Health Psychol..

[44] Shoji K., Cieslak R., Smoktunowicz E., Rogala A., Benight C.C., Luszczynska A. (2016). Associations between job burnout and self-efficacy: A meta-analysis. Anxiety Stress Coping..

[45] Schwarzer R., Hallum S. (2008). Perceived teacher self-efficacy as a predictor of job stress and burnout: Mediation analyses. Appl. Psychol..

[46] Jeung D., Kim C., Chang S. (2018). Emotional labor and burnout: A review of the literature. Yonsei Med. J..

[47] Skaalvik E.M., Skaalvik S. (2007). Dimensions of teacher self-efficacy and relations with strain factors, perceived collective teacher efficacy, and teacher burnout. J. Educ. Psychol..

[48] VanYperen N.W. (1998). Informational support, equity and burnout: The moderating effect of self-efficacy. J. Occup. Health Psychol..

[49] Jimmieson N.L. (2000). Employee reactions to behavioural control under conditions of stress: The moderating role of self-efficacy. Work Stress..

[50] Hsieh C., Hsieh J., Huang I. (2016). Self-efficacy as a mediator and moderator between emotional labor and job satisfaction: A case study of public service employees in Taiwan. Public Perform. Manag. Rev..

[51] Hackman J.R., Oldham G.R. (1975). Development of the job diagnostic survey. J. Appl. Psychol..

[52] Jackson S.E., Schwab R.L., Schuler R.S. (1986). Toward an understanding of the burnout phenomenon. J. Appl. Psychol..

[53] Han K., Shin C., Yoon H., Ko Y., Kim Y., Han C. (2018). Emotional labor and depressive mood in service and sales workers: Interactions with gender and job autonomy. Psychiatry Res..

[54] Humphrey R.H., Ashforth B.E., Diefendorff J.M. (2015). The bright side of emotional labor. J. Organ. Behav..

[55] Day A., Crown S.N., Ivany M. (2017). Organisational change and employee burnout: The moderating effects of support and job control. Saf. Sci..

[56] Jawahar I.M., Stone T.H., Kisamore J.L. (2007). Role conflict and burnout: The direct and moderating effects of political skill and perceived organizational support on burnout dimensions. Int. J. Stress Manag..

[57] Schaufeli W.B., Taris T.W., Bauer G., Hammig O. (2014). A critical review of the job demands-resources model: Implications for improving work and health. Bridging Occupational, Organizational and Public Health.

[58] Bakker A.B., Demerouti E., Taris T.W., Schaufeli W.B., Schreurs P.J.G. (2003). A multigroup analysis of the job demands-resources model in four home care organizations. Int. J. Stress Manag..

[59] Brotheridge C.M., Lee R.T. On the dimensionality of emotional labour: Development and validation of the emotional labour scale. Proceedings of the First Conference on Emotions in Organizational Life.

[60] Lee J., Han E., Hong H., Lee I. (2016). Validation of the Korean version of the Emotional Labor Scale (ELS). Korean J. Health Psychol..

[61] Shin K.H. (2003). The Maslach Burnout Inventory-General Survey (MBI-GS): An Application in South Korea. Korean J. Ind. Organ. Psychol..

[62] Rizzo J.R., House R.J., Lirtzman S.I. (1970). Role conflict and ambiguity in complex organizations. Adm. Sci. Q..

[63] Leiter M.P., Maslach C. (2000). Preventing Burnout and Building Engagement: A Complete Program for Organizational Renewal.

[64] Shim D., Ha S. (2013). A study on the relationship between job characteristics and innovative behavior: The mediating effect of self-efficacy. J. Ind. Innov..

[65] Breaugh J.A. (1999). Further investigation of the work autonomy scales: Two studies. J. Bus. Psychol..

[66] Grandey A.A. (1999). The Effects of Emotional Labor: Employee Attitudes, Stress and Performance. Ph.D. Thesis.

[67] Dormann C., Zapf D. (2004). Customer-related social stressors and burnout. J. Occup. Health Psychol..

[68] Podsakoff P.M., Organ D.W. (1986). Self-reports in organizational research: Problems and prospects. J. Manag..

[69] Ahola K., Honkonen T., Isometsä E., Kalimo R., Nykyri E., Koskinen S., Aromaa A., Lönnqvist J. (2006). Burnout in the general population. results from the Finnish health 2000 study. Soc. Psychiatry Psychiatr. Epidemiol..

[70] Gold Y. (1985). Does teacher burnout begin with student teaching. Education.

[71] Maslach C., Jackson S.E. (1985). The role of sex and family variables in burnout. Sex Roles.

[72] Baron R.M., Kenny D.A. (1986). The moderator—Mediator variable distinction in social psychological research: Conceptual, strategic, and statistical considerations. J. Pers. Soc. Psychol..

[73] Bakker A.B., Demerouti E. (2007). The job demands-resources model: State of the art. J. Manag. Psychol..

[74] Springer A., Oleksa K. (2017). The relationship between emotional labor and professional burnout: A comparative analysis between work of teachers and employees of commercial service sector. Med. Pr..

[75] Grandey A.A., Melloy R.C. (2017). The state of the heart: Emotional labor as emotion regulation reviewed and revised. J. Occup. Health Psychol..

[76] Wang G., Seibert S.E., Boles T.L., Charmine E.J.H., Ashkanasy N.M., Zerbe W.J. (2011). Chapter 1 Synthesizing what we know and looking ahead: A meta-analytical review of 30 years of emotional labor research. What Have We Learned? Ten Years On (Research on Emotion in Organizations, Volume 7).

[77] Gross J.J. (2002). Emotion regulation: Affective, cognitive, and social consequences. Psychophysiology.

[78] Zhao J.L., Li X.H., Shields J. (2018). Managing job burnout: The effects of emotion-regulation ability, emotional labor, and positive and negative affect at work. Int. J. Stress Manag..

[79] Gross J.J. (1998). The emerging field of emotion regulation: An integrative review. Rev. Gen. Psychol..

[80] Andela M., Truchot D., Borteyrou X. (2015). Emotional labour and burnout: Some methodological considerations and refinements. Can. J. Behav. Sci..

[81] Pandey J. (2018). Managing emotional labor for service employees: An HRM-based approach. Hum. Resour. Manag. Int. Dig..

[82] Ashkanasy N.M., Daus C.S. (2002). Emotion in the workplace: The new challenge for managers. Acad. Manag. Perspect..

[83] Drach-Zahavy A. (2010). How does service workers’ behavior affect their health? Service climate as a moderator in the service behavior-health relationships. J. Occup. Health Psychol..

[84] Le Blanc P.M., Hox J.J., Schaufeli W.B., Taris T.W. (2007). Take care! The evaluation of a team-based burnout intervention program for oncology care providers. J. Appl. Psychol..

[85] Grandey A.A., Foo S.C., Groth M., Goodwin R.E. (2012). Free to be you and me: A climate of authenticity alleviates burnout from emotional labor. J. Occup. Health Psychol..

[86] Maslach C., Jackson S.E. (1986). Maslach Burnout Inventory.

